# Blood Transcriptome Analysis of Beef Cow with Different Parity Revealed Candidate Genes and Gene Networks Regulating the Postpartum Diseases

**DOI:** 10.3390/genes13091671

**Published:** 2022-09-19

**Authors:** Yanda Yang, Chencheng Chang, Batu Baiyin, Zaixia Liu, Lili Guo, Le Zhou, Bin Liu, Caixia Shi, Wenguang Zhang

**Affiliations:** 1 College of Animal Science, Inner Mongolia Agricultural University, Hohhot 010010, China; 2 Inner Mongolia Mongol BioNew Technology Co., Ltd., Hohhot 010010, China; 3 College of Life Science, Inner Mongolia Agricultural University, Hohhot 010010, China

**Keywords:** Japanese black cattle, maternal parity, postpartum disease, transcriptome

## Abstract

Maternal parity is an important physiological factor influencing beef cow reproductive performance. However, there are few studies on the influence of different calving periods on early growth and postpartum diseases. Here, we conducted blood transcriptomic analysis on cows of different parities for gene discovery. We used Short Time Series Expression Miner (STEM) analysis to determine gene expression levels in cows of various parities and divided multiple parities into three main periods (nulliparous, primiparous, and multiparous) for subsequent analysis. Furthermore, the top 15,000 genes with the lowest median absolute deviation (MAD) were used to build a co-expression network using weighted correlation network analysis (WGCNA), and six independent modules were identified. Combing with Exon Wide Selection Signature (EWSS) and protein-protein interaction (PPI) analysis revealed that TPCN2, KIF22, MICAL3, RUNX2, PDE4A, TESK2, GPM6A, POLR1A, and KLHL6 involved in early growth and postpartum diseases. The GO and KEGG enrichment showed that the Parathyroid hormone synthesis, secretion, and action pathway and stem cell differentiation function-related pathways were enriched. Collectively, our study revealed candidate genes and gene networks regulating the early growth and postpartum diseases and provided new insights into the potential mechanism of reproduction advantages of different parity selection.

## 1. Introduction

Beef cow reproductive performance is a key factor affecting a ranch. It has a positive effect on stabilizing and improving the reproductive performance of beef cows [1] through the introduction of excellent varieties [2], strengthening disease prevention, and adopting some advanced technologies [3]. In fact, maternal parity is one of the important physiological factors affecting the reproductive performance of beef cows.

Calving is the basic factor for the profit of beef cow production [4]. Under the premise of meeting the basic frequency of calving and the quality of calving [5], the management plan for calving cows with different parity should be different. For example, primiparous cows need more nutrients to maintain their own development, and multiparous cows have higher postpartum morbidity and need prior prevention [6]. Predicting the risk of postpartum disease and evaluating the calving quality of different parity cows can effectively prolong the profit period of the beef cow [7,8]; reducing the number of feeding heads of reserve cows and the cost of semen consumption and herd renewal [9,10]; in the case of constant herd size, allowing pastures to carry out a higher proportion of active elimination [11], increasing the intensity of selection [12], thereby accelerating the genetic progress of herds [13], and the cost of ranching operations and veterinary drugs is also reduced [14].

Japanese black cattle are well-known for producing high-quality beef with a high intramuscular (marbling) fat content, which has been improved through genetic selection over the last half-century [15]. Previous research on Japanese black cattle has concentrated on genetic traits and management strategies to increase high-quality beef yields [7,16,17]. In fact, the effect of cattle genetic improvement through breeding will be more far-reaching and lasting, but it is slower than that of management decision-making. Appropriate breeding decision-making schemes can maximize the potential of herds in a limited time, improve economic efficiency, and accelerate genetic advance. In this study, we look at RNA-seq transcriptome profiles to find candidate genes in the blood of beef cows of various parities. We believe that these findings will aid in our understanding of the molecular mechanisms of early growth and development, as well as postpartum diseases in beef cattle of various parities, and will serve as a reference for decision-making programs for reproductive advantages of cows of various parities.

## 2. Materials and Methods

### 2.1. Laboratory Animals and Feeding Management

A total of 50 Japanese Black cattle individuals from different periods were used in this study (Supplementary Table S1). During their time at Inner Mongolia Mengdelong Dairy Farm, the cows were raised under similar feeding strategies and conditions (Hohhot City, Inner Mongolia Province). There were no restrictions on the food and drink given to the animals in this experiment. They were all raised in the same batch and fed the same diet.

### 2.2. Sequencing Data Analysis

Adaptors, raw reads with over 5% unknown nucleotides, and other low-quality reads with Q20 or lower quality scores were removed from raw reads to obtain high-quality clean reads. Subsequently, the clean data were mapped to the reference genome (Bos taurus ARS-UCD1.2) by the HISAT2 (v2.2.1, https://daehwankimlab.github.io/hisat2/, accessed on 5 May 2022) [18]. The alignment of effective reads with gene regions was calculated by using genomic location information from bovine reference genomes. SAMtools (v1.9, http://samtools.sourceforge.net/, accessed on 15 May 2022) was used to sort the BAM alignment files that were generated from HISAT2 by name. StrigTie (v2.1.1, https://ccb.jhu.edu/software/stringtie/index.shtml/, accessed on 15 May 2022) was used to estimate read counts and normalize reads as FPKM (fragments per kilobase of exon model per million mapped fragments) for each sample [19,20].

### 2.3. Short Time-Series Expression Miner Analysis

Short time-series expression miner (STEM) analysis is a well-validated and widely used bioinformatics approach that identifies statistically significant time-dependent gene expression profiles and identifies significantly enriched biological pathways within elucidated profiles [21]. An initial step of STEM is to select a distinct set of temporal model profiles that represent potential time-dependent gene expression patterns.

### 2.4. Differentially Expressed Genes Analysis

DESeq2 (v1.30.1, http://mirrors.nju.edu.cn/bioconductor/3.15/bioc/html/DESeq2.html/, accessed on 15 May 2022) was used to normalize the gene count data and calculate differential expression to investigate differentially expressed genes (DEGs) across three time periods (nulliparous vs. primiparous, primiparous vs. multiparous, nulliparous vs. multiparous). The DEGs were identified using DESeq2 and filtered using the *p*-value 0.05 criterion.

### 2.5. Co-Expression Network Analysis

WGCNA could be used to investigate gene-gene relationship patterns and measure the correlation between modules and the targeted traits, which has become a popular method for identifying candidate biomarkers. The function was used in the current study to perform hierarchical clustering based on DEG expression levels to reduce the effect of outlier samples on network analysis by per kilobases per million reads (FPKM). Using the TBtools R package, the top 15,000 genes with the lowest MAD were used to build a co-expression network [22]. When the fit index was 0.8, an appropriate value for the scale-free network construction was determined. The topological overlap matrix (TOM) for network interconnectedness was then calculated using the average linkage hierarchical clustering method. Using a dynamic tree-cutting algorithm for module division, with a minimum number of genes in each module of at least 40 and a threshold of 0.25 for similar module merging. Furthermore, we identified stage-specific modules with strong correlations between GS and MM values (*p*-value < 0.05) and highly correlated module-trait relationships (correlation coefficient > 0.5) [23].

### 2.6. Function Enrichment and PPI Analysis

For each stage-specific module, GO enrichment and KEGG pathway analyses were conducted using DAVID (https://david.ncifcrf.gov/, accessed on 20 May 2022) and KOBAS (http://kobas.cbi.pku.edu.cn/kobas3/, accessed on 20 May 2022). GO terms and pathways with a *p*-value ≤ 0.05 were defined as significantly enriched [24]. Genes in each stage-specific module were calculated separately by STRING (https://string-db.org/, accessed on 20 May 2022) and Metascape (https://metascape.org/, accessed on 20 May 2022), obtained the protein–protein interaction (PPI) network and imported into Cytoscape (v3.8.2, https://cytoscape.org/, accessed on 20 May 2022) [25,26]. Node sizes and colors indicate different node degrees, the width of the edges indicates combined scores and intra-module connection weights, and nodes with the largest size and red indicate the highest potential genes.

### 2.7. Exon Wide Selection Signature

The association analysis at the transcriptome level was used to assist in the screening of related important functional genes. The number of SNPs in the coding region, although small, is due to any base change in the exon. They may affect the translation of proteins and the expression of biological traits. Therefore, SNP is of great significance in the study of trait expression and disease. Exon Wide Selection Signature is an SNP screening method based on transcription level by detecting the index of population differentiation. The Fst is for screening selection signals among populations and then finding out the selection signal region and target traits related to important genes. The population differentiation index was calculated by the vcftools program, and the loci with negative Fst were eliminated to screen SNP selection signals of different parity beef cows (Fst > 0.15).

## 3. Results

### 3.1. Data Analysis of Transcriptome

**Figure 1:**
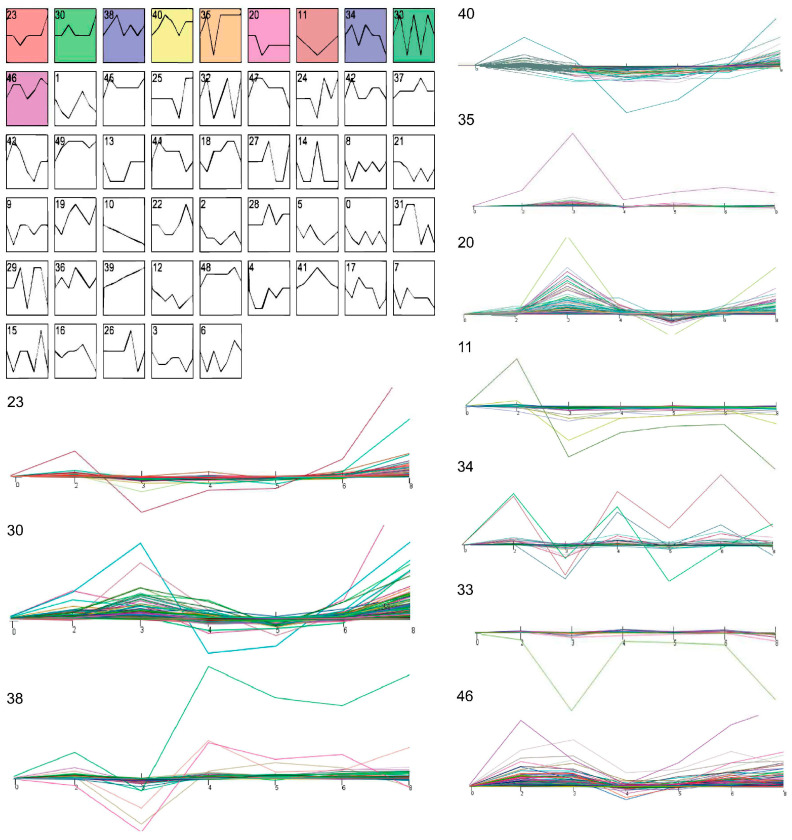
STEM analysis identified ten significant chronological profiles in gene expression, which are depicted graphically.

**Figure 2:**
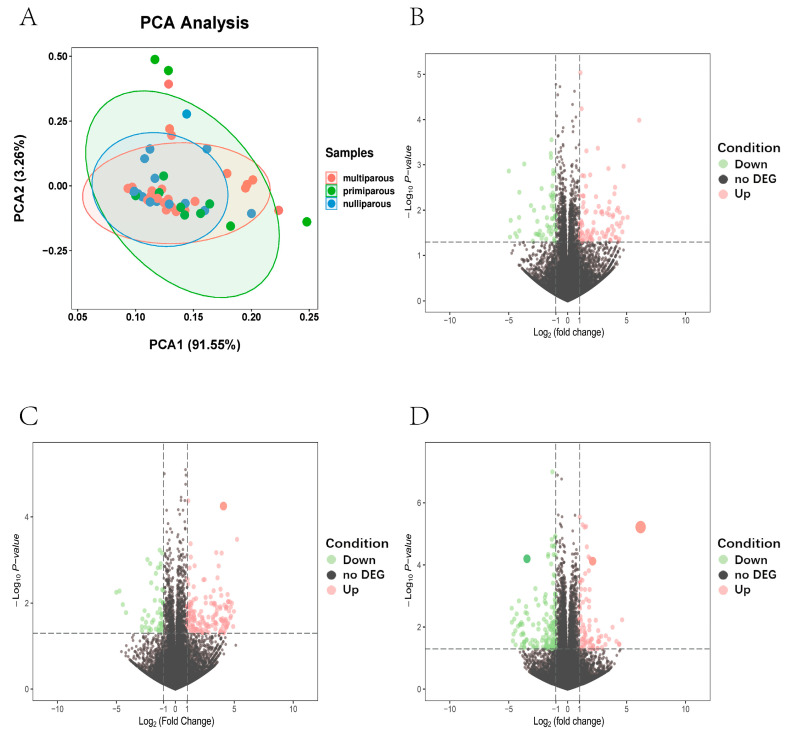
(**A**) PCA of the identified genes, the red, green, and blue dots represent samples of multiparous, primiparous, and nulliparous periods. (**B**) Volcano plot of differential genes, volcano plot for DEGs in the nulliparous period compared with the primiparous period. (**C**) Volcano plot for DEGs in the primiparous period compared with the multiparous period. (**D**) Volcano plot for DEGs in the nulliparous period compared with the multiparous period, red and green dots represent up/down-regulated DEGs, respectively. The gray dots represent not DEGs.

A total of 1246 million clean reads from 50 samples were generated by RNA-seq. After aligning clean reads to the cattle reference genome (ARS-UCD1.2), the mapping rate was approximately 95.35% (ranging from 93.73% to 96.13%) in Supplementary Table S1. We observed 24,577 genes that were expressed across 50 samples in Supplementary Table S2. By stem analysis, we found that gene expression changed greatly at 4 parities and divided all beef cows into three main stages: nulliparous, primiparous, and multiparous (Figure 1). In spite of differences in sample characteristics, gene expression profiles clustered samples from the same group (Figure 2A).

### 3.2. Differentially Expressed Genes across Three Periods

The researchers looked into the differences in gene expression of samples from different time periods. 1010 DEGs were identified in the primiparous vs. nulliparous comparison, including 457 upregulated genes and 553 downregulated genes (Figure 2B and Supplementary Table S3). In the multiparous vs. primiparous comparison, a total of 1060 DEGs were identified, including 616 upregulated genes and 444 downregulated genes (Figure 2C and Supplementary Table S4). In the multiparous vs. nulliparous comparison, 2245 DEGs were found, with 1182 upregulated genes and 1063 downregulated genes (Figure 2D and Supplementary Table S5).

### 3.3. Co-Expression Network Construction and Module Detection

**Figure 3:**
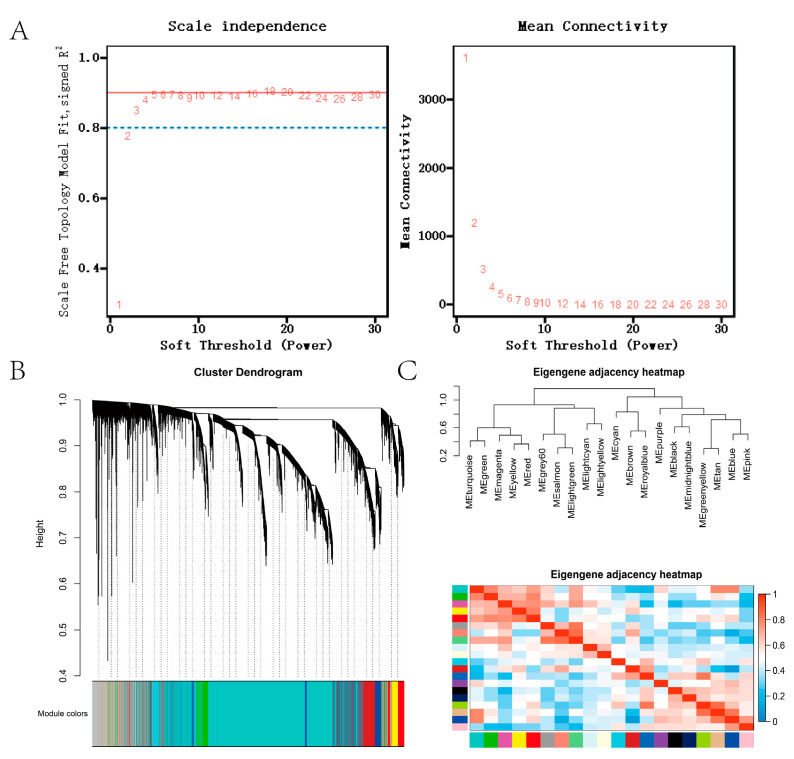
Scale independence of co-expression and Mean connectivity of co-expression. (**A**) Screening of soft thresholds. (**B**) Cluster dendrogram. (**C**). Eigengene dendrogram of modules and Eigengene adjacency heatmap.

We used WGCNA to investigate the relationship between three time periods to understand the function of genes better. According to the results of scale independence and mean connectivity, we took power as 5 when the correlation index reached 0.85 (Figure 3A) and 15,000 genes were divided into 19 modules (Figure 3B,C). The scale-free network was built with a block-wide module function, with 51 genes in the light yellow module and 1439 in the purple module.

### 3.4. Period-Specific Module Identification

**Figure 4:**
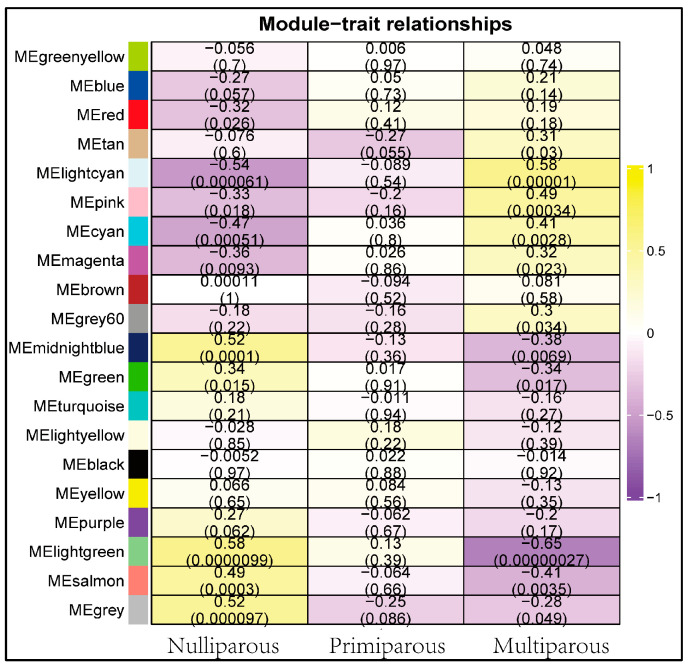
The correlation between the differentiation period and the modules. Correlation ranges from negative to positive according to the color, starting with purple and ending with yellow.

To investigate the period-specific modules, the GS and MM of all genes in the module were calculated over three periods. According to our definitions, GS is the correlation between a gene and its development period, and MM is the correlation between a gene’s expression profile and its module. A strong correlation between GS and MM (*p*-value < 0.05) demonstrates that genes strongly associated with a trait are frequently the most important components of the modules associated with that trait. Finally, we found six period-specific modules (average module-trait relationship > 0.5 and *p* < 0.05), of which the midnight blue, light green, and grey modules were positively correlated with the nulliparous period and the light cyan module was positively correlated with a multiparous period. In contrast, the module’s light cyan was negatively correlated with a nulliparous period, and the module’s light green was negatively correlated with the multiparous period (Figure 4).

### 3.5. Function Enrichment Analysis

**Figure 5:**
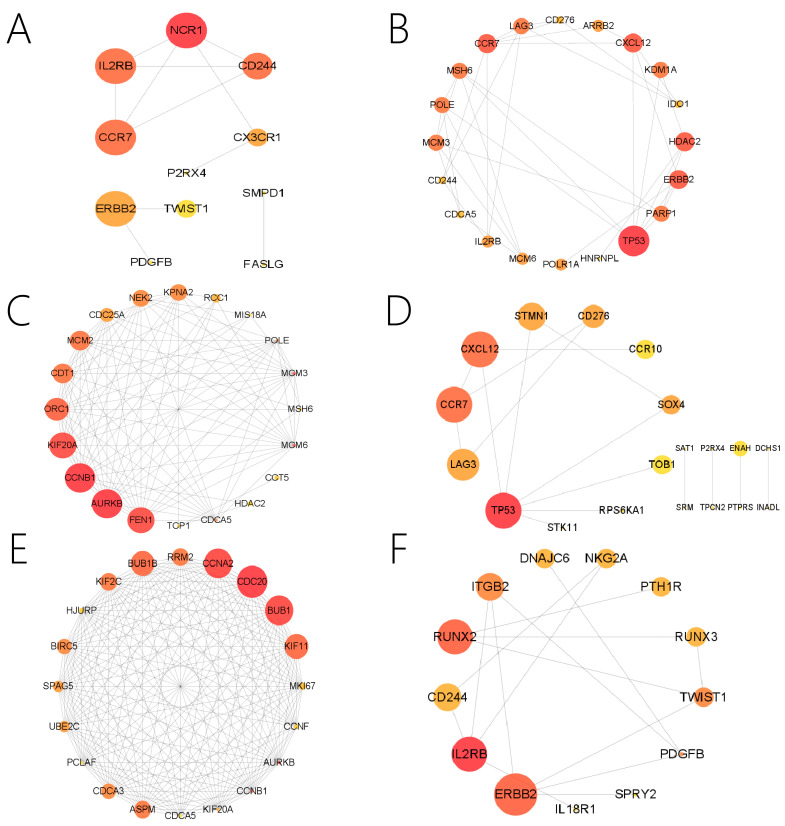
Visualization of potential genes in stage-specific modules. (**A**) PPI network for N-light cyan module. (**B**) PPI network for N-midnight blue module. (**C**) PPI network for N-light green module. (**D**) PPI network for N-grey module. (**E**) PPI network for M-light green module. (**F**) PPI network for M-light cyan module.

**Figure 6:**
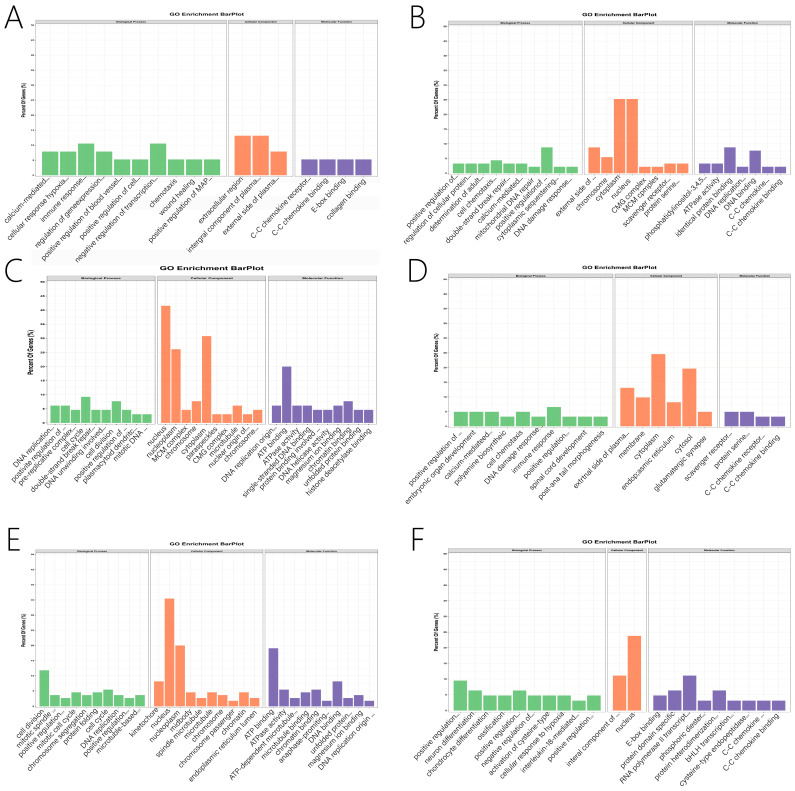
Analysis of gene ontology (GO) enrichment in related Specific module genes, the green part represents BP (Biological Process), the orange part represents CC (Cellular Component), and the purple part represents MF (Molecular Function). (**A**) GO enrichment analysis of genes in N-light cyan module. (**B**) GO enrichment analysis of genes in N-midnight blue module. (**C**) GO enrichment analysis of genes in N-light green module. (**D**) GO enrichment analysis of genes in the N-grey module. (**E**) GO enrichment analysis of genes in M-light green module. (**F**) GO enrichment analysis of genes in M-light cyan module.

**Figure 7:**
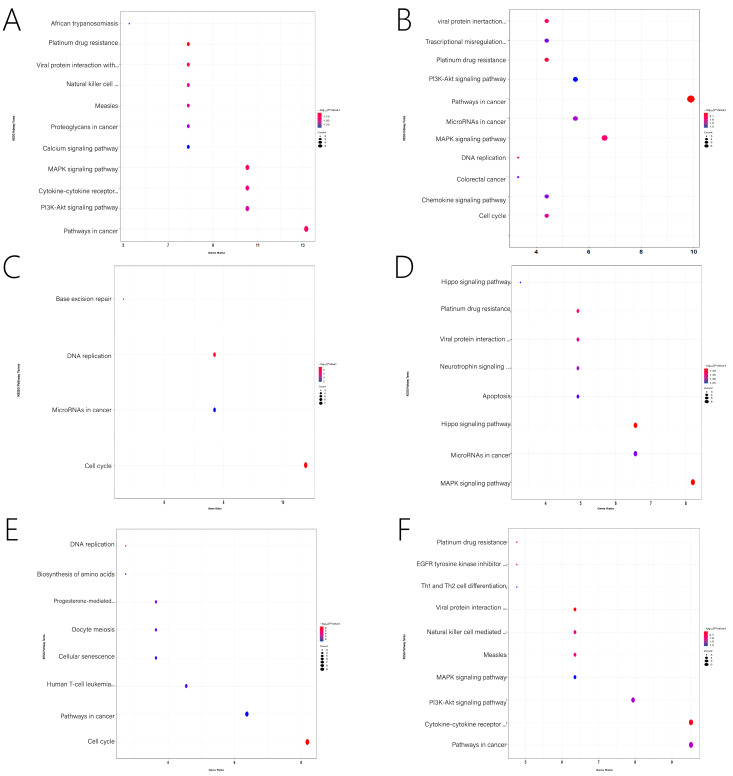
KEGG pathway analysis of related Specific module genes. (**A**) The pathway enrichment for the N-light cyan module. (**B**) The pathway enrichment for N-midnight blue module. (**C**) The pathway enrichment for N-light green module. (**D**) The pathway enrichment for the N-grey module. (**E**) The pathway enrichment for M-light green module. (**F**) The pathway enrichment for the M-light cyan module.

PPI network and Metascape were used to identify potential genes, and Cytoscape was used to visualize them. We present the significantly enriched GO terms and the pathways associated with each module in Figure 5. Detailed information on the GO terms and pathways is shown in Figure 6 and Figure 7 and Supplementary Table S7. For this study, the important pathways identified were Pathways in cancer, MAPK signaling pathway, MicroRNAs in cancer, PI3K-Akt signaling pathway, Platinum drug resistance, Cell cycle, Chemokine signaling pathway, Cancer-related transcriptional dysregulation Hippo signaling pathway, Progesterone-mediated oocyte maturation, cytokine-cytokine receptor interaction. Multiple significant GO terms are related to cell cycle, positive regulation of cell proliferation, cell division, mitotic cell cycle, immune response, neuron differentiation, positive regulation of ERK1 and ERK2 cascade, positive regulation of gene expression, mitotic spindle assembly checkpoint, chromosome segregation, DNA replication.

### 3.6. Exon Wide Selection Signature

**Figure 8:**
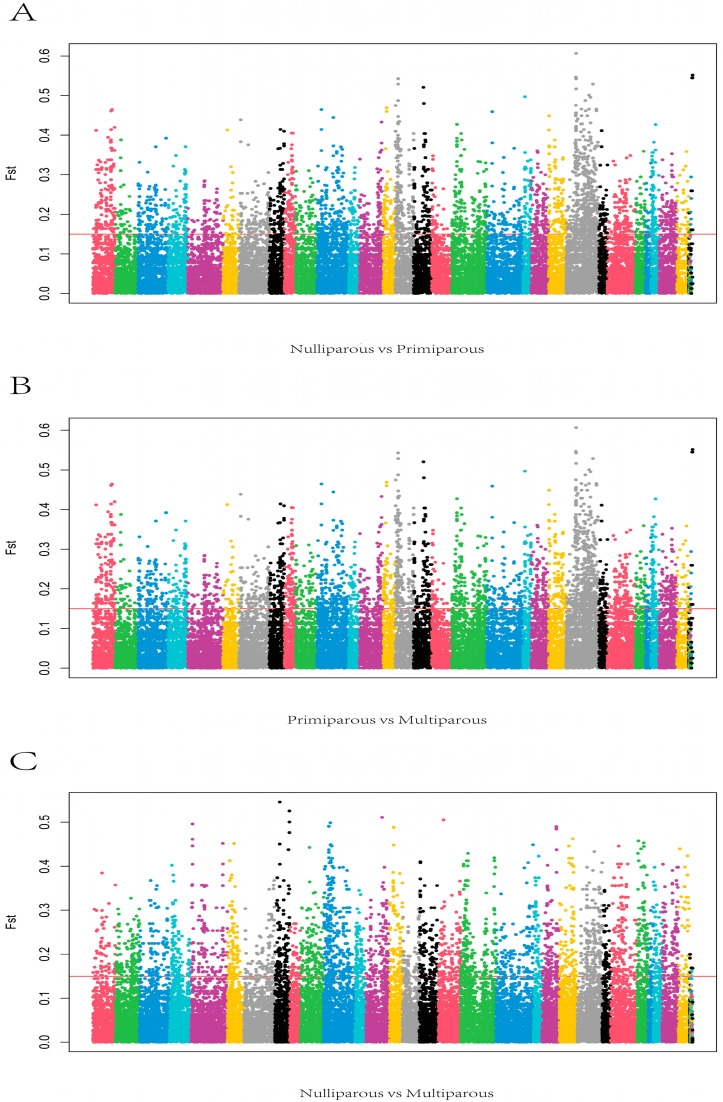
(**A**) Manhattan plots SPs in nulliparous compared with primiparous period (**B**) Manhattan plots SPs in primiparous compared with the multiparous period. (**C**) Manhattan plots SPs in nulliparous compared with the multiparous period.

In this study, SAMtools and BCFtools software were used to screen the SNP sites of cows with different parity. Based on the population differentiation index Fst, the differences of each genetic variation and its frequency between groups were compared, and the highly differentiated SNP sites were analyzed. SNP variation sites were screened by SAMtools and BCFtools. We screened 2541, 2744, and 1763 SNPs loci with high differentiation in different parity (Figure 8). Then, the selected SNP was mapped to the reference genome (Bos taurus ARS-UCD1.2), with highly differentiated SNP regions as the center, and the 1M extensions in the upstream and downstream were used as new candidate regions. Finally, we mapped 723, 945, and 548 genes over three periods (Supplementary Table S6).

### 3.7. Top Genes Expressed in Three Periods

**Figure 9:**
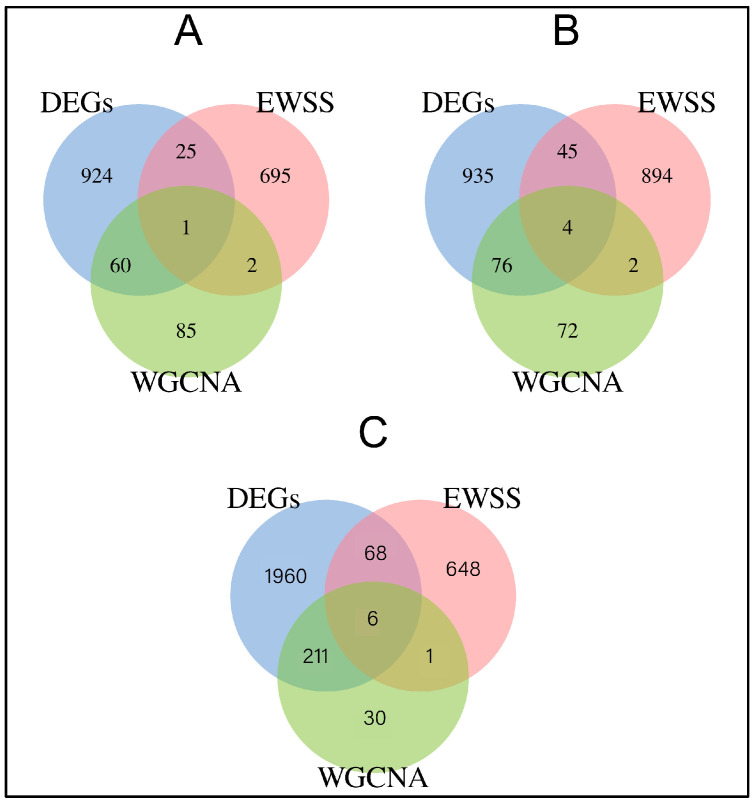
Venn diagram of DEGs, EWSS, and WGCNA. (**A**) The common intersection of nulliparous and primiparous groups under the DEGs, EWSS, and WGCNA analysis method. (**B**) The common intersection of primiparous and multiparous groups under the DEGs, EWSS, and WGCNA analysis method. (**C**) The common intersection of nulliparous and multiparous groups under the DEGs, EWSS, and WGCNA analysis method.

In this study, we found that in the comparison of the nulliparous vs. primiparous group, it was mainly concentrated in the signal pathways such as Oxidative phosphorylation, Pathways of neurodegeneration-multiple diseases, and Thermogenesis. In the primiparous vs. multiparous group, it is mainly concentrated in signaling pathways such as postpartum disease, such as Salmonella infection, AMPK signaling pathway, Transcriptional misregulation in cancer, and the Insulin signaling pathway. This is highly consistent with our previous post-partum disease plate phenomenon. Based on the DEG, WGCNA, and EWSS results, we can suggest TPCN2, KIF22, MICAL3, RUNX2, PDE4A, TESK2, GPM6A, POLR1A, and KLHL6 as the promising candidate genes for different parity cows (Figure 9).

## 4. Discussion

In the livestock industry, reproductive traits are important economic traits, and they are influenced by both genetics and the environment. In this experiment, beef cows are all raised in the same environment. In this study, we found some special genes, which are TPCN2 in the nulliparous vs. primiparous group; KIF22, MICAL3, RUNX2, and PDE4A in the primiparous vs. multiparous group; TESK2, PDE4A, GPM6A, POLR1A, KLHL6 and RUNX2 in nulliparous vs. multiparous group. Both RUNX2 and PDE4A were specifically expressed in groups primiparous and multiparous, and they work together on the Parathyroid hormone synthesis, secretion, and action signaling pathway. The Parathyroid hormone synthesis, secretion, and action-signaling pathway were demonstrated to be involved in translation initiation and translation process, which is critical in the growth, metabolism, reproduction, and aging of organisms [27].

RUNX2 is located on chromosome 6, also known as CCD or AML3. Runt DNA-binding domain-containing nuclear protein encoded by this gene is a member of the RUNX family of transcription factors. This protein is required for osteoblast differentiation and skeletal morphogenesis, as well as acting as a scaffold for nucleic acids and regulatory factors involved in skeletal gene expression. This gene has also been associated with carcass and growth-related traits in broilers [28] and goats [29]. According to some findings, RUNX2 may play an oncogenic role in esophageal carcinoma by activating the PI3K/AKT and ERK pathways [30]. PDE4A, also known as DPDE2 or PDE46, is located on chromosome 19. The protein encoded by this gene belongs to the cyclic nucleotide phosphodiesterase (PDE) family and PDE4 subfamily. This PDE hydrolyzes the second messenger, cAMP, which is a regulator and mediator of a number of cellular responses to extracellular signals. PDE4 inhibitors might be useful therapeutic targets for myelodysplastic syndromes (MDS) [31]. T-cells that were PDE4A-transgenic were also partially protected from regulatory T-cell suppression [32]. Furthermore, PDE4A effectively suppressed PGE2-mediated upregulation of the inhibitory surface markers CD73 and CD94 on CD8 T-cells [33].

In the nulliparous vs. primiparous group, we identified only one gene. TPCN2, also known as TPC2 or SHEP10, is located on chromosome 11. TPCN2 protects against diet-induced weight gain in a mild manner, and this protection is likely independent of glucose tolerance, insulin sensitivity, and fasting plasma and hepatic lipid levels [34]. The endolysosomal two-pore cation channel TPCN2 is a key factor in neovascularization and immune activation [35]. TPCN2 acts on autophagy progression and extracellular vesicle transport in cancer cells [36]. The encoded protein is a catalytic subunit of the complex that transcribes DNA into ribosomal RNA precursors. Tp53-dependent neuroepithelial apoptosis decreased neural crest cell proliferation, and cranioskeletal abnormalities can all result [37]. KIF22 on chromosome 11, also known as OBP or KNSL4. This gene encodes a protein that belongs to the kinesin-like protein family and plays an important role in metaphase chromosome alignment and maintenance. KIF22 may be involved in the regulation of cell proliferation in colon cancer [38]. In addition, it is highly expressed in pancreatic cancer; it regulates the MEK/ERK/P21 signaling axis and promotes the cell cycle and the development of pancreatic cancer [39]. MICAL3 on chromosome 22 is mainly involved in activating actin-binding activity and actin filament depolymerization. MICAL3 knockout results in an increased frequency of cytokinetic failure and delayed abscission. MICAL3 directs adaptor protein ELKS and Rab8A-positive vesicles to the midbody via a mechanism unrelated to its enzymatic activity, and ELKS and Rab8A deficiency causes cytokinesis defects [40].

The KLHL6 gene on chromosome 3 encodes a protein that is involved in B-lymphocyte antigen receptor signaling and germinal-center B-cell maturation [41]. TESK2 is a serine/threonine protein kinase with an N-terminal protein kinase domain that is structurally similar to the kinase domains of testis-specific protein kinase-1 and LIM motif-containing protein kinases [42]. GPM6A, also known as M6A, is located on chromosome 11. It is predicted to enable calcium channel activity and is involved in neuron migration and stem cell differentiation. POLR1A is also known as A190, RPA1, and RPO14 on chromosome 2. The protein encoded by this gene is the largest subunit of the RNA polymerase I complex. The encoded protein is a catalytic subunit of the complex that transcribes DNA into ribosomal RNA precursors. Tp53-dependent neuroepithelial apoptosis decreased neural crest cell proliferation, and cranioskeletal abnormalities can all result [43].

## 5. Conclusions

In this study, blood transcriptome analysis was performed on different parities of Japanese black cattle. DEGs, EWSS, and WGCNA analysis revealed that TPCN2, KIF22, MICAL3, KLHL6, and POLR1A might be involved in candidate genes for postpartum diseases. RUNX2, PDE4A, TESK2, and GPM6A genes could be used as candidate genes for early growth development. It provides a theoretical basis for beef cattle breeding evaluation.

## Data Availability

The data presented in this study are available on request from the corresponding author.

## Supplementary Materials

The following supporting information can be downloaded at: https://www.mdpi.com/article/10.3390/genes13091671/s1, Table S1: Number of reads, and number of aligned reads, per cattle sample; Table S2: Gene expression matrix; Table S3: Number of differentially expressed genes between primiparous and nulliparous stages; Table S4: Number of differentially expressed genes between multiparous and primiparous stages; Table S5: Number of differentially expressed genes between multiparous and nulliparous stages; Table S6: Number of EWSS mapped genes in different periods; Table S7: Significant module enrichment.
